# Genetic identification of cytomegaloviruses in a rural population of Côte d’Ivoire

**DOI:** 10.1186/s12985-015-0394-1

**Published:** 2015-10-05

**Authors:** Augustin Etile Anoh, Chantal Akoua-Koffi, Emmanuel Couacy-Hymann, Maude Pauly, Grit Schubert, Arsène Mossoun, Sabrina Weiss, Siv Aina J. Leendertz, Michael A. Jarvis, Fabian H. Leendertz, Bernhard Ehlers

**Affiliations:** Centre de Recherche pour le Développement, Université Alassane Ouattara de Bouake, 01 BP V18 Bouake, Côte d’Ivoire; LANADA/Laboratoire Central de Pathologie Animale, Bingerville, 206 Côte d’Ivoire; Project group P3 “Epidemiology of Highly Pathogenic Microorganisms”, Robert Koch Institute, Berlin, 13353 Germany; Division 12 “Measles, Mumps, Rubella and Viruses affecting immune-compromised patients”, Robert Koch Institute, Berlin, 13353 Germany; Present address: Department of Infection and Immunity, Luxembourg Institute of Health, Esch-sur-Alzette, 4354 Luxembourg; UFR Biosciences, Université FHB, Abidjan-Cocody, Côte d’Ivoire, Abidjan, Côte d’Ivoire; Present address: European Public Health Microbiology (EUPHEM) training programme, European Centre for Disease Prevention and Control (ECDC), Stockholm, Sweden, and Public Health England (PHE), London, NW9 5EQ UK; School of Biomedical and Healthcare Sciences, Plymouth University, Plymouth, United Kingdom

**Keywords:** Herpesvirus, Cytomegalovirus, Glycoprotein B, Côte d’Ivoire, Human, Colobus, Monkey, Zoonosis, Bushmeat

## Abstract

**Background:**

Cytomegaloviruses (CMVs) are herpesviruses that infect many mammalian species, including humans. Infection generally passes undetected, but the virus can cause serious disease in individuals with impaired immune function. Human CMV (HCMV) is circulating with high seroprevalence (60–100 %) on all continents. However, little information is available on HCMV genoprevalence and genetic diversity in subsaharan Africa, especially in rural areas of West Africa that are at high risk of human-to-human HCMV transmission. In addition, there is a potential for zoonotic spillover of pathogens through bushmeat hunting and handling in these areas as shown for various retroviruses. Although HCMV and nonhuman CMVs are regarded as species-specific, potential human infection with CMVs of non-human primate (NHP) origin, shown to circulate in the local NHP population, has not been studied.

**Findings:**

Analysis of 657 human oral swabs and fecal samples collected from 518 individuals living in 8 villages of Côte d’Ivoire with generic PCR for identification of human and NHP CMVs revealed shedding of HCMV in 2.5 % of the individuals. Determination of glycoprotein B sequences showed identity with strains Towne, AD169 and Toledo, respectively. NHP CMV sequences were not detected.

**Conclusions:**

HCMV is actively circulating in a proportion of the rural Côte d’Ivoire human population with circulating strains being closely related to those previously identified in non-African countries. The lack of NHP CMVs in human populations in an environment conducive to cross-species infection supports zoonotic transmission of CMVs to humans being at most a rare event.

## Findings

Human cytomegalovirus (HCMV) is a ubiquitous herpesvirus (subfamily *Betaherpesvirinae)* that infects the majority of the human population by early adulthood [1]. Although generally benign in healthy individuals, HCMV can cause serious disease in the absence of competent immune function, such as occurs in newborns, non-HAART treated AIDS patients and transplant recipients undergoing iatrogenic immunosuppression [2–4]. Similar to all herpesviruses, acute infection by HCMV and nonhuman CMV is followed by establishment of a persistent/latent infection for the lifespan of the host, with periodic reactivation and shedding. Superinfection of HCMV seropositive individuals is also possible, resulting in the frequent circulation of multiple HCMV strains within the population. Although regarded as highly species-specific, the capacity for zoonotic transmission of nonhuman CMV from closely related nonhuman primate (NHP) wildlife species remains an important, but unexplored question. HCMV sero- and genoprevalence and strain sequences have been determined in several countries world-wide (e.g. [5–11]), but little information is available from subsaharan Africa [12–18], especially on CMV nucleotide sequences in rural areas of West Africa that are at high risk of human-to-human HCMV transmission and are zoonotic ‘hot-spot’ regions due to behaviors such as bush-meat hunting and slaughtering [19–21].

A landmark study by Jones et al. [22] has identified emerging infectious disease (EID) ‘hotspots’ within poorer regions of West Africa, South America and Asia that are most frequently associated with zoonotic emergence of pathogens with global health significance. In the present study, human subjects in rural Côte d’Ivoire living in villages surrounding the Taï Forest National Park were analyzed for shedding of HCMV and NHP CMVs. NHPs are a primary source of zoonotic disease [23–27] and wildlife including monkeys represents an important component of diet (“bush meat”) in rural Côte d’Ivoire [28–30]. A recent study has shown a considerable incidence of CMV in colobus monkeys from this geographic region, 10 % in black-and-white colobus and 22 % in western red colobus [31]. This rural human study group therefore represented a population with potentially high exposure to NHPs carrying and excreting NHP CMVs. We reasoned that focusing on the identification and characterization of CMVs within those individuals actively shedding CMV within this large human study population would provide a sensitive means by which to assess on the one hand the circulating HCMV strains and on the other hand the propensity for zoonotic transmission of NHP CMVs.

**Fig. 1:**
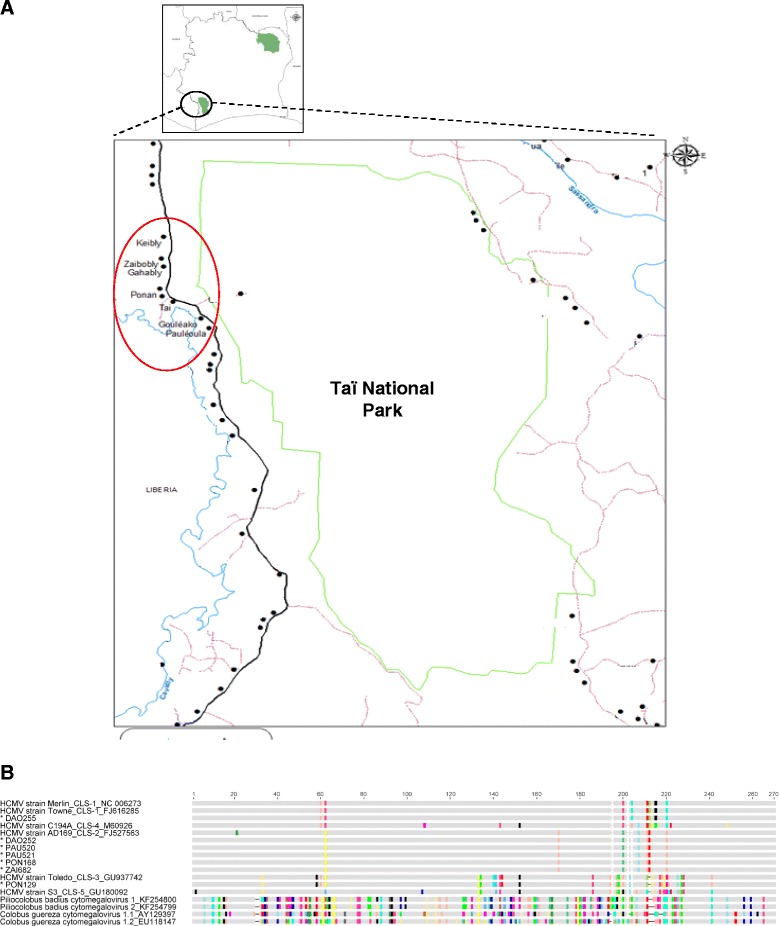
**a** Map of the study area in Western Côte d’Ivoire showing the sampling localities in the red circle. Villages are represented by black dots, the boundary of Taï National park by green line, road by black line, tracks by purple lines, rivers by blue lines. **b** Alignment of partial glycoprotein B amino acid (*aa*) sequences shown as a cartoon. Conserved aa are given in grey color; non-conserved aa are colored according to their type. The region between aa positions 190 and 230 comprises the recognition motif for the cellular endoprotease furin. For the HCMV strains, their cleavage site (*CLS*) type is indicated by numbers (*CLS1-CLS5*). The study sequence names are marked with a star

As part of a larger study investigating human contact to animal viruses through bush meat hunting, preparation and consumption [32–34], 657 samples (472 oral swabs and 185 fecal samples) from 518 apparently healthy human subjects were included in the present investigation. The study underwent ethics review and approval (permit number 101-10/MSHP/CENR/P; Abidjan, Côte d’Ivoire), and its purpose was explained to district health authorities and villagers prior to sample collection. Following informed consent and completion of questionnaires aimed at collecting general demographic data and determining exposure to NHP-derived bushmeat, samples were collected between May and October 2012 in 8 villages situated close to the boundary of the Taï National park (Fig. 1a), an area with documented transmission of zoonotic viruses from non-human primates to humans [19]. DNA was extracted from oral swabs with the QIAamp blood & tissue kit and from fecal samples with the Roboklon stool kit according to manufacturer’s instructions, and DNA was stored at −20 °C.

For the detection of HCMV and NHP CMVs, generic PCRs were performed targeting either CMV glycoprotein B (gB; UL55) (PCR 1) or UL56 (PCR 2) coding sequences (CDS) using two nested degenerate primer sets. These had been designed earlier to enable detection of both HCMV strains and HCMV-related animal betaherpesviruses and were performed here as described previously (see PCR 1 and PCR 2 in reference [31]). Using these primers, several NHP CMVs and rodent CMVs had been discovered previously [31, 35, 36]. Longer gB sequences of identified HCMVs were obtained by applying nested non-degenerate (specific) primers (PCR 3), with amplification products of 1.06 kb in first round and 0.9 kb in second round. The forward and backward primers of the first round were 5’- CGTTACGTGACGGTCAAGGA −3’and 5’- GTGAGTAGCAGCGTCCTGG −3’, respectively; those of the second round 5’- GCACCTGGCTCTATCGTGAG −3’and 5’- GCGGCAATCGGTTTGTTGTA −3’. The first round was performed with 5μl of DNA as template, 12.7μl of PCR mix (containing 1× AmpliTaq™ buffer, 2.0 mM MgCl_2_, 5 % DMSO, dNTPs at 200 mM each, 1.0 unit of AmpliTaq Gold Polymerase; Applied Biosystems) and 1 μM of each first-round sense and antisense primer. In the second-round amplification, 1 μl of the first amplification product was added to 12.7 μl of PCR mix with 1μM of each second-round primer. PCR was performed on a T-gradient S thermocycler (Biometra, Germany) with ‘hot-start’ activation of polymerase at 95 °C for 12 min, followed by 45 cycles of denaturation (95 °C, 30s), annealing (60 °C, 30s), and elongation (72 °C, 2min), and final elongation at 72 °C for 10min. In addition, for comprehensive amplification of colobus CMVs, a nested set of non-degenerate (specific) primers (PCR 4) was used that targets binding sites completely conserved among all publicly available gB nucleic acid sequences of colobus CMVs (but not HCMVs) (see PCR 4 in ref. [31]).

With PCR 1 (target: gB CDS) and PCR 2 (target: UL56 CDS) HCMV was identified in 16 oral and fecal samples of 13 individuals (7 oral and 2 fecal samples with PCR 1; 4 oral and 3 fecal samples with PCR 2). This equates to an overall detection rate (combined results of PCR 1 and 2) of 2.6 % in the samples and 2.5 % (CI 1.43–4.29 %) in the human subjects. None of the subjects was positive in both oral and fecal samples. In BLAST analysis, the Ivorian HCMV sequences were 99–100 % identical to known HCMV types. Colobus CMV sequences were not detected.

Since the amplified HCMV sequences were rather short for conclusive comparison with published sequences, we amplified an extended gB sequence (approximately 0.9 kb) using PCR 3 that covers the region of highest amino acid diversity within the gB gene, surrounding the R-X-K/R-R recognition motif for the cellular endoprotease furin [37], and allows to readily differentiate the detected HCMVs from each other and from colobus CMVs. This was successfull for 7 individuals (from villages Daobly, Gouliako, Pauleoula, Ponan, Taï, and Zaïpobly; see Fig. 1a and b). The sequences were deposited in GenBank under the accession numbers KT716432- KT716438. In a nucleotide alignment these sequences were all 99–100 % identical to known HCMVs (strain Merlin, Genbank accession number [acc. no.] NC006273; strain Towne, acc. no. FJ616285; strain Toledo, acc. no. GU937742; strain AD169, acc. no. FJ527563) (Fig. 1b). In contrast, colobus CMV DNA was not detected in a large subset (306 oral and 160 fecal) of the above described samples (107 oral and 25 fecal samples were exhausted) using colobus CMV-specific PCR 4 (CI 0.00-0.01 %), while control PCR performed using colobus monkey samples that had been previously determined to contain colobus CMV genomic DNA [31], showed consistent detection of colobus CMV by all PCRs (PCRs 1, 2, 4). A recent study has shown a considerable prevalence of CMV in black-and-white (10 %) and western red (22 %) colobus monkeys from this geographic region [31], and the exposure rate to colobus monkeys through hunting, slaughtering and preparation of bushmeat around the Taï National Park is high [29, 38]. Taking this into account, our results suggest that colobus CMVs are not frequently transmitted to humans. The results do not necessarily imply that CMV transmission from monkeys to humans is completely absent, but the relatively large number of human samples collected over 8 villages suggests that if transmission does occur, it is an extremely rare event that does not commonly result in establishment of a persistent infection. It is also possible that individuals infected with colobus CMV may have remained unidentified because the virus was not shed in saliva and feces or because the applied PCRs did not possess maximum sensitivity for detection of CMVs. Despite this, the 2.6 % HCMV prevalence determined here in a collection of oral and fecal swabs is well in accord with previous PCR-based studies on healthy subjects reporting HCMV detection rates in blood of 0–8 % of children and adults from Uganda, Germany, Latvia, Australia and Japan [39–43] (studies based on fecal samples were to our knowledge not reported). In addition, sequences of other viruses have been detected previously in the present sample set [33] indicating absence of PCR inhibitors, and the use of colobus-specific PCR able to identify colobus CMV in control colobus monkey samples indicates this PCR is of sufficient sensitivity to detect colobus CMV present at levels found within its natural host. Taken together, this suggests that, if individuals were infected with colobus CMV, absence of shedding in feces and saliva or inhibition of PCR detection was likely not a critical confounding factor.

The likely absence of monkey-to-human cross-species transmission in the setting investigated here and our previous finding that monkey CMVs are not transmitted to chimpanzees [31], indicates a high level of specificity of CMVs to their primate hosts, with absence of a capacity for infection in closely related primate species. This suggests that the potential for environmental spread of CMVs from target to non-target host species is generally low and that CMV, similar to other DNA viruses [44], are inable to genetically adapt to a level sufficient to infect and then persist within the new host. Uncharacterized protective host mechanisms may also play a role [45], as *in vitro* studies showed that species-specific CMV replicated only poorly in cells from other species [46, 47]. Taken together, these factors may severely limit the potential of the virus for adaptation to a new host and the ability for cross-species epizootic/zoonotic transmission [31].

There is a high burden of disease due to HCMV infection in newborns, non-HAART treated AIDS patients and transplant recipients. Several vaccines are presently under development, among them a glycoprotein B-based vaccine [48]. HCMV strains are highly variable and naturally acquired HCMV infection does not produce immunity to reinfection with a different strain. Therefore, knowledge of worldwide HCMV diversity is of critical importance for further vaccine development. The sequence data obtained here from people in rural Côte d’Ivoire suggest that strain diversity does not substantially differ from that in other parts of the world. Determination of complete genome sequences would be desirable to confirm this finding.
